# Use of medical tourism for hip and knee surgery in osteoarthritis: a qualitative examination of distinctive attitudinal characteristics among Canadian patients

**DOI:** 10.1186/1472-6963-12-417

**Published:** 2012-11-21

**Authors:** Valorie A Crooks, Keri Cameron, Vera Chouinard, Rory Johnston, Jeremy Snyder, Victoria Casey

**Affiliations:** 1 Department of Geography, Simon Fraser University, 8888 University Dr, Burnaby, BC, V5A 1S6, Canada; 2 School of Geography and Earth Sciences, McMaster University, 1280 Main Street West, Hamilton, ON, L8S 4L8, Canada; 3 Faculty of Health Sciences, Simon Fraser University, 8888 University Dr, Burnaby, BC, V5A 1S6, Canada

## Abstract

**Background:**

Medical tourism is the term that describes patients’ international travel with the intention of seeking medical treatment. Some medical tourists go abroad for orthopaedic surgeries, including hip and knee resurfacing and replacement. In this article we examine the findings of interviews with Canadian medical tourists who went abroad for such surgeries to determine what is distinctive about their attitudes when compared to existing qualitative research findings about patients’ decision-making in and experiences of these same procedures in their home countries.

**Methods:**

Fourteen Canadian medical tourists participated in semi-structured phone interviews, all of whom had gone abroad for hip or knee surgery to treat osteoarthritis. Transcripts were coded and thematically analysed, which involved comparing emerging findings to those in the existing qualitative literature on hip and knee surgery.

**Results:**

Three distinctive attitudinal characteristics among participants were identified when interview themes were compared to findings in the existing qualitative research on hip and knee surgery in osteoarthritis. These attitudinal characteristics were that the medical tourists we spoke with were: (1) comfortable health-related decision-makers; (2) unwavering in their views about procedure necessity and urgency; and (3) firm in their desires to maintain active lives.

**Conclusions:**

Compared to other patients reported on in the existing qualitative hip and knee surgery literature, medical tourists are less likely to question their need for surgery and are particularly active in their pursuit of surgical intervention. They are also comfortable with taking control of health-related decisions. Future research is needed to identify motivators behind patients’ pursuit of care abroad, determine if the attitudinal characteristics identified here hold true for other patient groups, and ascertain the impact of these attitudinal characteristics on surgical outcomes. Arthritis care providers can use the attitudinal characteristics identified here to better advise osteoarthritis patients who are considering seeking care abroad.

## Background

Medical tourism is the term used to describe patients’ intentional travel across national borders for medical care [1,2]. This self-initiated travel does not involve ill or injured vacationers, nor those seeking care in another country through an established cross-border care arrangement (i.e., an arrangement between health insurers in typically proximal countries that enables patients to access care in another country through this insurance plan) [1,3,4]. Triggered by patients’ desires to access medical care that is not affordable, not available either at all or in a timely fashion, and/or not legal in their home countries, the global medical tourism industry has grown significantly in the past decade [1,5]. Worldwide, people travel to access private medical care from hospitals actively seeking to attract international patients, many of which are in lower- and middle-income countries [6,7]. Canadian patients, who serve as the focus of this article, are among those going abroad as medical tourists [8-10].

Orthopaedic surgeries, including hip replacement and resurfacing and knee replacement (HRRKR), are one of the procedures sought by international patients at medical tourism hospitals [1,11-13]. While such procedures are commonly offered in patients’ home health care systems, they may simply be more accessible, affordable, or available elsewhere. Although Canadian patients have access to a public Medicare system that provides them with necessary medical care with no out-of-pocket costs, including orthopaedic surgeries for which they are referred, some of these patients choose to exit the domestic system and pay privately to access care abroad [10]. For these patients, it is commonly speculated that long wait-times for surgical procedures in the public system drive them abroad [9,10,14,15].

As the Canadian population continues to age, rates of musculoskeletal disease such as osteoarthritis are increasing, in turn increasing demand for orthopaedic surgeries [16-19]. Meanwhile, system capacity has not always kept up with procedure demand [18,20], which has left some patients with debilitating osteoarthritis on wait-lists for HRRKR surgeries for extended periods of time [21-23]. For example, the national benchmark for obtaining hip replacement is 26 weeks (there are no benchmarks for hip resurfacing as it has limited domestic capacity due primarily to lack of surgical expertise). In 2010, 84% of Canadians obtained a hip replacement within the benchmark period [24]. This varied between provinces, with only 57% of those wait-listed in Nova Scotia obtaining surgery within this period versus 91% of patients in Ontario [24]. The national benchmark for obtaining knee replacement is also 26 weeks. In 2010, 79% of Canadians in need of this procedure obtained surgery within the benchmark period, with inter-provincial variance ranging from 42-89% [24]. Certainly, wait-times are not the sole driver of Canadians’ pursuit of HRRKR surgeries abroad to treat osteoarthritis [10], but they do offer important context for understanding trends among patients in countries with public health care systems who choose to pay out-of-pocket for care elsewhere.

Dedicated attention has yet to be given to the issue of osteoarthritis patients’ travel abroad for HRRKR, due at least in part to the fact that the field of medical tourism research is young and much remains to be learned [1,6,25,26]. The potentially altered or heightened health risks that patients face traveling abroad for surgery, such as contracting post-operative infections, having inadequate access to safe drugs or blood supplies, or developing deep vein thrombosis at the time of surgery or while flying due to lessened mobility or atmospheric pressure changes, mean that those in the arthritis care community need to be attentive to the issue of osteoarthritis patients’ travel abroad for surgery [1]. The need for this attentiveness is heightened given the potential for arthritis care providers to be involved in offering patient education about medical tourism [8]. There also may be a need for patients’ home health care systems to provide follow-up care once patients return from surgery abroad [8]. Health care providers, including arthritis care professionals, and administrators must thus be aware of and plan for the needs of HRRKR patients upon return home [1,8]. This includes being prepared to treat post-operative complications [12]. The provision of post-operative care at home for medical tourists is a particularly complicated situation given that some physicians have expressed reluctance to treat complications or offer follow-up care for patients who have privately-pursued medical care in other countries due to liability concerns, wherein destination country facilities, procedures, quality standards, and surgeons may be unknown to domestic providers [1,6].

In this article we present the findings of an exploratory qualitative study examining 14 Canadian patients’ use of medical tourism for HRRKR to treat osteoarthritis. In an attempt to better understand what, if anything, is distinctive about osteoarthritis patients who choose medical tourism versus those who remain in their home countries, we conducted a thematic analysis that compared and contrasted distinctive attitudinal characteristics (i.e., commonalities in these patients’ attitudes or mindsets towards seeking out and obtaining HRRKR surgeries) among the medical tourists we interviewed to reported findings in the existing *qualitative* literature about patients’ decision-making for and experiences of these surgeries in their home countries. Understanding attitudinal characteristics is of great value as they directly shape patients’ beliefs, decisions, and actions regarding treatment, compliance, information sharing with physicians, and conceptualizations of risk and so need to be understood by those in clinical practice in order to enable person-focused care and realize a therapeutic relationship [27-34]. The existing qualitative HRRKR literature offers several important insights for understanding attitudinal characteristics among this patient group, including that: not all those recommended for HRRKR surgeries choose to obtain them [34-39]; some patients see osteoarthritis as a normal part of aging and thus do not view surgical intervention as necessary, preferring less invasive options such as weight loss and using walking aids or no treatment at all [37-42]; and some osteoarthritis patients do not want the responsibility of making the final decision for whether or not HRRKR surgery happens [41,43]. We use these and other findings from existing qualitative studies to come to a better understanding of what, if anything, is distinctive about osteoarthritis patients who choose to access HRRKR abroad with the intent of identifying implications for arthritis care practice and future research.

## Methods

This analysis is part of an exploratory instructive case study we, a team of health services researchers trained in the social and health sciences, undertook to examine how Canadians decide to go abroad as medical tourists. As research in this field is at a nascent stage [1,6], we came to this exploratory study with no existing expectations regarding patients’ decision-making in medical tourism. Part of our study involved interviewing Canadians who had previously gone abroad as medical tourists. This article examines the experiences of 14 such patients, namely those who went abroad for HRRKR. Ethical approval for the study was obtained from the Office of Research Ethics at Simon Fraser University prior to data collection. All participants signed consent forms prior to participating and their anonymity is protected.

### Recruitment

We recruited Canadian medical tourists to participate in semi-structured phone interviews through purposive and snowball sampling. The inclusion criteria for participation were that participants: (1) were over the age of 18; (2) had intentionally accessed surgery in another country that was not part of a cross-border care arrangement; and (3) were Canadian citizens or permanent residents. Multiple recruitment strategies were used simultaneously. Of the 14 participants, five were recruited through industry contacts (i.e., using medical tourism brokers, agents who make bookings for international patients), five through public media reports or online testimonials containing patients’ names from which we found their contact information in public directories, three through snowballing, and one through advertisements placed in newspapers and online forums. Those interested in participating contacted a research assistant who confirmed their eligibility, after which an interview was scheduled. Nobody who met the inclusion criteria and scheduled an interview withdrew from the study.

### Data collection

Phone interviews were undertaken over a six month period in 2010. As many potential participants as could be identified were contacted during this period. Data collection ceased once the six-month period ended. All interviews were conducted by the fourth author. Interviews lasted for approximately 1.5 hours and were conducted only after a signed consent form was received. Interviews were semi-structured, meaning that participants were able to discuss things they thought were relevant but had not been explicitly asked about [44]. Interviews were organized using a guide developed in an iterative process by the first, fourth, and fifth authors after a thorough review of the medical tourism literature. The guide probed: (1) health status; (2) logistics of care abroad; (3) experiences abroad; (4) decision-making; and (5) ethical considerations. There were 29 questions across these five areas, most of which had additional sub-questions that were used to further probe each issue. Table 1 contains selected questions from the guide, illustrating the types of issues inquired about. 

**Table 1:** Selected questions from semi-structured interview guide

**Interview Guide Section**	**Selected Questions**
Health status	●How would you characterize your overall state of health? ●Are you presently under the care of any specialists?
Logistics of care abroad	●When was it that you traveled to __________________ for the procedure? ●Did you have any concerns about recovering from the procedure away from home? What were they?
Experiences abroad	●What are some of the key differences between health care here and ____________? Why? ●Did you have any complications after your surgery or complications from your surgery? What were they? Where were they taken care of (Canada or abroad)?
Decision-making	●What concerns, if any, did you have about traveling to _____________ for _________ procedure? ●Why did you decide to get _________________ done?
Ethical considerations	●Did your plan to go abroad for surgery raise any ethical concerns for you? ●Did other people raise any ethical concerns regarding your trip and surgery?

A limitation of our data collection strategy is that interviews were only conducted in English. There were, however, no requests made by potential participants to be interviewed in French or other languages. Another limitation is that interviews were conducted by phone. Phone interviewing limits observations of participants’ surroundings and body language. However, these interviews are becoming increasingly common because they yield high-quality data and are cost effective [45]. As many participants were located thousands of kilometres away from the interviewer, contact by phone was what enabled data collection.

### Data analysis

Interviews were digitally recorded and transcribed verbatim. Transcripts were entered into N7 (a qualitative data management program), after which coding that incorporated inductive and deductive codes was undertaken in several steps. First, a preliminary scheme was created by the first, fourth, and fifth authors following independent transcript review and several group discussions. Second, the fourth author and an external researcher not affiliated with the study separately coded two transcripts using the draft scheme. The input of this external researcher was sought to confirm the reliability of the scheme and the parameters of each code [46]. Following this, the scheme was revised by the fourth author using input from the external researcher, wherein redundant or underused codes were collapsed or eliminated and overused codes were disaggregated. Once they reached consensus on the revised scheme it was shared with the first and fifth authors, who were involved in its initial development, to ensure agreement on the changes. Third, the fourth author applied the revised coding scheme to the full dataset. Throughout these steps notes were kept in order to establish an audit trail in order to heighten trustworthiness [46].

Following coding, codes related to the analytic focus on ‘attitudinal characteristics’ were extracted from the dataset and shared with all authors in preparation for thematic analysis. These extracts were independently reviewed by all authors in order to identify analytic themes by observing patterns and outliers in the data [47]. Following independent review, meetings were held in which individual and collective interpretations of emergent themes were discussed and also compared to the existing qualitative literature on HRRKR surgeries. Our use of investigator triangulation at this point and throughout the coding and analysis processes was purposefully done in order to enhance rigour.

Comparing emerging findings in an ongoing study to established findings in the existing literature is an important component of thematic analysis [46,47]. It was through this process of comparing the themes identified from our coded interview data to existing findings in the literature that we came to identify what appear to be distinctive attitudinal characteristics of Canadians with osteoarthritis who go abroad for HRRKR surgeries when compared to qualitative research-based characterizations of patients who have been recommended for such surgeries and stay in their home countries either to have or not have a procedure. Three such attitudinal characteristics were identified through our iterative process of review and discussion, which were agreed upon by all authors. These are expanded upon in the following section. Quotes that best illustrate important dimensions of these attitudinal characteristics were selected by the lead author for inclusion here based upon input from all investigators in team meetings and feedback throughout the writing process.

## Results

Fourteen Canadians, eight women and six men, with osteoarthritis who had gone abroad for HRRKR surgeries in the last eight years were interviewed. Three participants had more than one procedure at different times, one had a bi-lateral replacement at the same time, and the remainder had one procedure, which means that 18 joint resurfacing or replacement procedures in total were obtained abroad by this group. Table 2 summarizes key participant variables. Eight traveled abroad with a friend or family member, and four used the services of a broker to assist with arranging their care. Participants’ average age when going abroad for surgery was 59.

**Table 2:** Participant overview

**Procedure**	**Destination**	**Year(s) of surgery**	**Age(s) at surgery**	**Sex**	Household income range*	Had traveled internationally before**	**Had been wait- listed in Canada**
Hip resurfacing (x 2)	India	2006, 2007	59, 60	F	>$80,000	Y	Y
Hip resurfacing	India	2009	49	F	>$80,000	Y	Y
Hip resurfacing	India	2005	63	M	$50,000 - $80,000	Y	N
Knee replacement (*x*2)	India	2008, 2009	54	F	$50,000 - $80,000	Y	Y
Hip resurfacing	India	2007	62	F	>$80,000	Y	Y
Knee replacement	Cuba	2007	57	F	>$80,000	N	Y
Hip resurfacing	India	2010	67	M	$50,000 - $80,000	Y	N
Hip resurfacing	India	2009	55	M	$50,000 - $80,000	Y	N
Hip replacement	Germany	2007	77	F	$50,000 - $80,000	Y	Y
Knee replacement	India	2010	62	M	<$30,000	N	N
Hip resurfacing (*x*2)	India	2008, 2010	62, 64	M	>$80,000	Y	N
Hip resurfacing	India	2004	42	M	$30,000 - $50,000	Y	Y
Bilateral knee replacement	India	2007	58	F	>$80,000	Y	Y
Hip resurfacing	India	2008	58	F	>$80,000	Y	N

* Canadian dollars.

** Travel to the continental United States has been excluded as this is rarely thought of as ‘international travel’ by Canadians.

In general, it was found that participants initiated their consideration of medical tourism for HRRKR surgery: after being placed on a wait-list that they thought was too long; over concern about being placed on a wait-list in the future that was *perceived* to be lengthy; or upon encountering a lack of access to a preferred procedure (typically hip resurfacing, which has limited availability in Canada). Despite these differing prompts, all participants indicated that, in retrospect, they were satisfied with their decisions to go abroad for HRRKR surgery. Only one indicated that he would not go abroad for surgery again, which was due to the fact that he developed a gastro-intestinal illness while recovering abroad.

In comparing Canadian medical tourists’ discussions of HRRKR in the interviews to those of other patients in the existing qualitative HRRKR literature using thematic analysis, three attitudinal characteristics consistently emerged as distinctive regarding medical tourists’ mindsets and beliefs towards surgery, in that they were particularly: (1) comfortable as health-related decision-makers; (2) unwavering in their views about procedure urgency and necessity; and (3) firm in their desires to maintain active lives. These attitudinal characteristics held true for participants regardless of differences among them in what prompted them to go abroad or the specific procedure sought abroad. In the remainder of this section we examine these three attitudinal characteristics in-depth. In the discussion section that follows we interpret these findings as they relate to the existing qualitative research on HRRKR surgery in order to highlight their distinctiveness.

### Comfortable health-related decision-makers

All 14 participants portrayed themselves as being very comfortable with making health-related decisions about engaging in medical tourism on their own, including doing so independent of their regular health care providers. This involved making decisions regarding procedure type, destination, surgeon, and affordability, among other factors. While there were many instances where participants sought the advice of a regular health care provider, they all chose to take decision-making matters into their own hands for HRRKR surgery abroad. For example, when one participant was asked if he had consulted with his family doctor about going to India for surgery prior to departing, he said: “*it was a very little…conversation about that. More or less I did most…everything…on my own*.” Another said almost the same thing, explaining that “*I just did it on my own*” when it came to learning about procedure and destination options and deciding to go to India for hip resurfacing. Meanwhile, four participants said they purposely did not tell their regular doctors about their plans to go abroad due to a perception that it fell outside their scope of practice or that they would be unsupportive: “*I knew her answer was going to be ‘oh what are you doing that for’*.”

Participants typically saw themselves as managers of their own medical cases, seeking information as needed from websites, friends and family, health care providers abroad, Canadian health care providers, and medical tourism brokers as needed in order to inform their decisions regarding medical tourism. Many reported information-gathering experiences such as this one: “*Through the internet I read quite a bit about…hip resurfacing and as it turned out one of my co-workers was talking about this quite a bit before I actually went…so he, we talked about it…I just read everything I could and mostly it was all on the internet.*” Particular value was placed on having details that were thought to be reliable about the reputations of surgeons abroad and hearing testimonials from other patients who had gone to the same facility. Having access to both of these types of information increased participants’ comfort with their attitudes towards having confidence in their decisions. A man who went to India for hip resurfacing told of how patient testimonials spread by word-of-mouth gave him comfort: “*there’s a large group of people from here that have gone to this exact surgeon…and have had a lot of success so that’s overriding I guess [for my] comfort.*” The attitudes of others sometimes reinforced participants taking on a manager role. For example, when talking about the possibility of going to India for surgery, a participant’s husband said ‘*It is up to you what you choose…it’s your body*.’ Such comments underscored participants’ need to take leadership over their health-related decision making and develop an attitude of comfort in doing so. In effect, by removing themselves from their usual health care system the participants were thrust into the role of patient case manager, which hinged on their comfort as self-advocates and sound decision-makers.

### Unwavering views about procedure urgency and necessity

The participants projected an attitude of being completely convinced of their need to undergo HRRKR surgery in order to minimize or eliminate pain and restore their quality of lives. As one woman who went to India twice for hip resurfacing explained: “*At that time [before going to India] I was just in so much pain that I wanted to get better*.” For the participants, their pre-surgery pain levels were viewed as neither tolerable nor a natural part of how they viewed their own aging process, wherein many indicated that they felt ‘too young’ to be dealing with the levels of pain and immobility they were experiencing. These pain levels were also viewed as a problem they had to proactively manage by necessity: “*I was 58 years old! I needed my life back, and I couldn't wait for the Canadian government to give it back to me.*” A woman who went to India for a knee replacement said:

"I needed to get the care for me. I’d waited long enough here. I knew I would get it there [India], and that was my main concern. And you know they, they took care of my needs immediately… That’s why I went over. My needs were taken care of right away."

As per this woman’s comment, convinced of the necessity of joint surgery, participants saw wait-lists as an impediment to better health outcomes, making the pursuit of private medical care abroad an appealing option. It is for this reason that some chose to go abroad before even being assigned to a wait-list in Canada, in that any waiting period was viewed as not desirable regardless of its length.

Participants’ unwavering views and attitudes regarding the necessity of surgery to treat their osteoarthritis drove them to identify ways to overcome potential barriers to their access to medical care abroad. For example, a woman who went to Germany for hip replacement minimized the potential barrier of out-of-pocket expenses, saying: “*it [going abroad] was simply a decision…did I want to sit here for another year or so [on a wait-list] not being able to move from the couch to the kitchen or with this great pain, or spend some money and be able to get around again?*” The potential for pain relief through surgery resulted in her using some retirement investment money to go abroad. This was common among participants, where most people drew on savings or personal loans so that cost did not prevent them from accessing surgery they felt was urgently needed. Language barriers also had to be addressed by some participants, including with regard to obtaining translations of paperwork and medical documentation. The participant who went to Cuba, for example, had no Spanish language competency and aside from one surgeon who spoke limited English found herself in a hospital where “*there wasn’t anybody that spoke any English.*” The potential for pain relief and a better quality of life in part justified getting medical care in destinations with limited English capacity, in that all participants indicated being satisfied with their decisions to go abroad for care and would recommend that others do the same regardless of language barriers.

### Desiring to maintain active lives

At the time they went abroad for surgery, six participants were employed. Many others had significant volunteer commitments and/or assisted with family businesses. Physical hobbies such as skiing, cycling, and hiking were also regularly discussed during interviews. For example, a man who went to India for hip resurfacing said this about his post-recovery life: “*My hip is as good as it was before I ever had any arthritis. I’ve never had any concerns from the day [of the surgery to] six weeks after, when I went downhill skiing. I don’t even think about it*.” Participants typically characterized themselves and their lives as active, or always “*on the go*” and adopted the attitude that surgery would enable them to return to their active lives, thereby further reinforcing the perceived necessity of surgical intervention. Prior to surgery abroad, osteoarthritis had limited their abilities to be active, including in relation to work productivity, in desired ways. The speed with which they could obtain surgery abroad made medical tourism an appealing option for regaining their active lives.

Through the process of researching medical tourism options, nine participants learned of hip resurfacing as a possible alternative to hip replacement. Their correspondence with surgeons abroad and with others who had undergone the procedure previously, often via online forums, led them to believe that having resurfacing instead of replacement would enable them to regain a more active lifestyle after surgery. A participant explained:

"I wanted to have total recovery in terms of being able to do everything I did before [developing osteoarthritis]… I am an aggressive athlete, and that I’m relatively young, I…decided that the procedure of total hip [replacement] was too radical because…just doing less is better than more. If I have a problem with my hip resurfacing I go to a total hip, if I have a problem with a total hip, I could be threatened with being crippled. "

Others reported adopting this same attitude towards opting for hip resurfacing abroad despite being wait-listed or being concerned about being wait-listed for hip replacement at home, with one referring to a total joint replacement as the “*old fashioned way of doing it.*” Their pursuit of this alternative surgery out of a desire to have a procedure that would enable greater activity levels upon recovery is further reflective of participants’ comfort with taking leadership over their health-related decisions and managing their medical care.

## Discussion

Through thematic analysis it was found that the Canadian medical tourists we spoke with consistently demonstrated three shared attitudinal characteristics regarding their mindsets and beliefs towards surgery, in that they were particularly: (1) comfortable as health-related decision-makers; (2) unwavering in their views about procedure urgency and necessity; and (3) firm in their desires to maintain active lives. In much of this discussion section we consider these findings in relation to the existing qualitative research on patients’ decision-making for and experiences of HRRKR surgeries. It is important to emphasize before doing so, however, that our contention is not that medical tourists are the only HRRKR patients who demonstrate these attitudinal characteristics. Instead, our key point is that the *confluence* of these three characteristics characterizes the mindsets of Canadian medical tourists who have sought HRRKR surgeries abroad when contrasted against published accounts shared in the qualitative HRRKR literature of patients deciding about or receiving such surgeries in their home countries.

When compared to the existing literature, the results of our thematic analysis show that medical tourists seem to be less likely to question the need for surgery or to regard osteoarthritis as a ‘normal’ part of the aging process versus the findings of other qualitative studies on HRRKR surgery [37-42]. There are a number of potential explanations for this, one of which is that our participants were relatively young and active when compared to those in other studies. For example, the age range of our study was 42 to 77, which closely resembles the age range 40–84 in Woolhead et al.’s [48] qualitative study of patients’ perceptions of the outcomes of knee replacement, but has many more ‘younger’ participants than the qualitative studies of Hamel et al. [40] and Gustafsson et al. [49], both of which had no participants under the age of 65. As such, the medical tourists we interviewed may have been less likely to accept osteoarthritis pain as an expected part of their life-stage and were thus more proactive in obtaining surgery in order to maintain active lives by virtue of their age. Others have commented on the importance of age to patients’ orthopaedic surgical decision-making. For example, Hawker [36] observes that younger patients are more willing to consider total joint replacement and Hudak et al. [37] contend that as people age there is an expectation of pain due to chronic illness and co-morbidities and this expectation has a relationship to patients’ willingness to obtain surgery.

The medical tourists we interviewed strongly desired surgical interventions, which is not in keeping with the findings of some other qualitative studies that have shown that particular patients with osteoarthritis can have low willingness to undergo joint surgery, sometimes resulting in them deciding to not obtain HRRKR [34-39]. This finding is perhaps not surprising as the participants we interviewed had already undergone surgery abroad and in doing so realized their strong desires for surgery, likely best reflecting the mindsets of participants in these other qualitative studies who most wanted surgical interventions and ultimately obtained them rather than those who wanted them least and did not. Specific motivations for seeking surgery were, however, revealed in our study. The strong desire among our participants to obtain surgery through taking charge of, and ultimately managing, their medical care is linked to their desires to regain a more active life. In fact, they actively worked at overcoming barriers to surgery, such as cost, which is consistent with Marcinkowski et al.’s [50] finding that motivated osteoarthritis patients will actively work to lessen surgical wait-times. In the case of medical tourists, this ultimately involves accessing medical care in a new country with an unfamiliar health care system, which is a high level of ‘active work’ when one considers that some osteoarthritis patients will not even consider changing local surgeons to lessen wait-times [16].

The participants of this study projected attitudes of comfort pertaining to deciding whether or not to obtain joint surgery, which country to pursue surgery in, and also which surgeon to use with minimal support from others, and particularly health care providers. Meanwhile, other qualitative studies have found that some patients recommended for joint surgery do not want to hold overall responsibility for making significant decisions [41,43]. Instead, they prefer to be involved in the decision-making process but do not want to be responsible for making the final decision as to whether or not surgery is obtained. Along these lines, while some patients awaiting HRRKR surgery are rather passive in waiting for information about surgery to be presented to them [50], our participants were active in seeking out information and becoming educated about their procedures, managing themselves throughout this process. Again, these findings serve to highlight the distinctiveness of the attitudinal characteristics of our participants when compared to findings in the existing qualitative literature on HRRKR.

### Implications for arthritis care and practice

We see three main implications of the findings of this analysis for arthritis care practice. First, it is important for arthritis care providers to be observant of attitudinal and other characteristics of patients who go abroad for surgery in their own practice so that they can identify, at an early stage, those who might benefit from receiving patient education regarding the risks and benefits of medical tourism. Given that several participants did not speak with their regular health care providers about their plans to go abroad for surgery, leaving it to patients to initiate such dialogue might not be an effective strategy. Such education could focus on discussing follow-up care plans, identifying the risks of pursuing medical care abroad, and encouraging patients to seek travel medicine advice. This might also provide an opportunity to discuss the importance of obtaining translated medical records from abroad so that procedure details can be shared with patients’ regular arthritis care providers, as this has been cited as a problem inherent to medical tourism [1].

Second, participants’ accounts reveal the importance of word-of-mouth and online networks among patients who have had HRRKR surgery abroad for those engaging in decision-making about medical tourism. Given the information-dissemination roles that former medical tourists play, arthritis care professionals may wish to engage in discussions with those who are in their practices about their experiences of going abroad and also whether or not they regularly or even sometimes speak with potential medical tourists. Talking with these former medical tourists about who, if anyone, they have talked with about about pursuing surgery abroad will assist arthritis care professionals with better understanding attitudinal and other trends among medical tourists and their information networks. It will also enable them to underscore the importance of having former medical tourists couch their personal experiences within an awareness of the overall potential risks and benefits of medical tourism when they do speak with those considering medical tourism. This can raise former medical tourists’ awareness of the responsibilities they hold towards the larger patient community.

Third, the findings of this study underscore the importance of acknowledging the roles that patients’ understanding of their own bodies, health and wellbeing, and aging play in their decision-making regarding HRRKR surgery. The people we spoke with often viewed their hip and/or knee impairments to be inconsistent with their age also lifecourse stage. This was one of the main drivers of their pursuit of care abroad, wherein they felt as though they were too young to wait long periods for surgery or that their lives were too active to have more invasive surgery than what they wanted for themselves. It would behoove arthritis care professionals to understand these types of viewpoints held by their patients, particularly when consulting with them about the potential of obtaining surgery. This is because arthritis care professionals have the capacity to tailor such pre-surgical consultations to patients’ specific needs and concerns and so are well positioned to assess concerns regarding a patient’s ability to maintain an active life following surgery, among others. Doing so may also enable arthritis care professionals to better anticipate which of their patients might consider going abroad for medical care and thereby with engaging in appropriate patient education, as discussed above in the first implication for practice.

### Wider relevance

Canadians are but one of the dozens of patient groups known to travel abroad to obtain surgeries in other countries via the medical tourism industry [1,2,4-6,12]. While we have focused on Canadian patients in this analysis, our findings are likely to be of relevance to the health systems of any country in which patients are traveling abroad for HRRKR, and especially in those countries where these procedures are funded through a public health care system. There is currently no basis in the international medical tourism literature for suggesting that patients in other countries would report radically different attitudes, although there is an acknowledgement that the factors that motivate patients to consider surgery abroad are often shaped by local context [1,6]. As we have pointed out above, an important implication for future research is to examine the attitudes of medical tourists in other countries towards their pursuit of these surgeries in order to understand whether or not the findings of this analysis hold true in these other contexts. Furthermore, arthritis care providers in any home country of medical tourists can benefit from being attentive to the pursuit of medical tourism by their osteoarthritis patients and from considering the impacts it has on their own practice, which is an implication stemming from the findings presented here.

### Future research directions

This analysis raises a number of implications for future research, four of which we highlight here. First, typical accounts of the motivations of Canadians seeking surgery abroad focused on the issue of excessive wait-times in the domestic system [9,14,15,51-53]. However, as is demonstrated by our study, procedure availability plays an important role in Canadians’ medical tourists’ decision-making for HRRKR surgery. Future research must be attentive to the variety of motivators that lie behind patients’ pursuit of care abroad.

Second, research is needed to determine if the distinctive attitudinal characteristics identified here adequately characterize other medical tourism patient groups, including those from other countries seeking HRRKR surgery and Canadians pursuing other procedures abroad. Along these lines, it would be useful to examine the perspectives of those who considered going abroad for HRRKR as medical tourists but decided not to, along with those who did go but were dissatisfied, in order to determine whether these groups hold distinctive characteristics of their own. Admittedly, these are difficult patient groups to locate and so the feasibility of pursuing such lines of research needs to be carefully considered.

Third, we need to understand how the distinctive attitudinal characteristics we have identified inform short and long-term surgical outcomes. For example, the participants’ relative independence from domestic care providers resulting from their enactment of a ‘case manager’ role may have implications for their abilities to access follow-up care or receive reliable pre-operative advice in their home systems. This is but one of the ways in which attitudinal characteristics that informed participants’ decisions to go abroad may also have long-term impacts on their health and wellbeing.

Fourth, further research is needed on all aspects of patients’ use of medical tourism for HRRKR surgery. For example, attention needs to be given to understanding the concerns that arthritis care providers have regarding their patients accessing surgery abroad, the frequency with which their own patients pursue care abroad, and the responsibilities they think they hold towards patients who opt for medical tourism. Both qualitative and quantitative studies to effectively address this knowledge gap, as well as the other three mentioned above.

### Limitations

In the methods section we identified some limitations of our study design. Another important limitation must be acknowledged. Although this is a qualitative study and thus does not seek generalizability, the lack of surveillance of outbound medical tourism by Canadians [1,8,26] limits our abilities to provide contextual information about how many Canadians go abroad for HRRKR, the full range of countries they visit, or the demographic characteristics that make up this group. We are thus unable to comment on how similar or dissimilar the participants in this study are to this patient group as a whole.

## Conclusions

In this article we have presented the findings of a thematic analysis examining attitudinal characteristics among 14 Canadian osteoarthritis patients who went abroad for HRRKR as medical tourists that were identified as distinctive when we compared emerging themes from the interviews to existing findings in the qualitative HRRKR literature. Our analysis identified three such distinctive attitudinal characteristics, in that the medical tourist participants were all: (1) comfortable in their abilities to make health-related decisions, often independent of their regular health care providers; (2) convinced of the urgency and necessity of obtaining joint surgery; and (3) firm in their desires to maintain active lives. Importantly, our contention is not that medical tourism patients are the *only* ones who hold such attitudinal characteristics. For example, some patients who obtain HRRKR surgery domestically may very well be independent decision-makers or want desperately to regain their active lives. Rather, it is that the confluence of all three attitudinal characteristics seems to characterize the mindsets of medical tourists specifically when their thoughts and experiences are contrasted against patients’ accounts in the existing qualitative literature on HRRKR surgeries. This contention, though supported by the findings of our thematic analysis, must be considered in light of the fact that this study is exploratory and the representativeness of the participants among all medical tourists is unknown due to a lack of population-level data. Thus, it is preliminary and requires further research in order to be assessed and confirmed in full.

## Abbreviations

HRRKR: Hip replacement and resurfacing and knee replacement.

## Competing interests

The authors declare that they have no competing interest.

## Authors’ contributions

VAC designed the study, led this analysis, and led writing this article. KC contributed to conceiving of this analysis, the process of confirming the interpretation of findings, and to drafting the discussion section. V. Chouinard contributed to conceiving of this analysis, the process of confirming the interpretation of findings, and editing this article. RJ conducted the interviews and made other contributions to study design, contributed to conceiving of this analysis, the process of confirming the interpretation of findings, and to drafting the discussion section. JS contributed to overall study design and edited this article. V. Casey contributed to the process of confirming the interpretation of findings and editing this article. All authors read and approved the final manuscript.

## Pre-publication history

The pre-publication history for this paper can be accessed here:

http://www.biomedcentral.com/1472-6963/12/417/prepub
